# A Rare Case of Coexisting Mutation in Desmin and Thioredoxin Reductase 2 Genes Causing Dilated Cardiomyopathy

**DOI:** 10.7759/cureus.40560

**Published:** 2023-06-17

**Authors:** Nazima Khatun, Sahil Zaveri, Louis Salciccioli, Sabu John

**Affiliations:** 1 Internal Medicine, State University of New York Downstate Medical Center, Brooklyn, USA; 2 Cardiovascular Disease, State University of New York Downstate Medical Center, Brooklyn, USA; 3 Cardiology, Kings County Hospital Center, Brooklyn, USA

**Keywords:** thioredoxin reductase 2, desmin, rare genetic mutation, heart failure with reduced ejection fraction, dilated cardiomyopathy (dcm)

## Abstract

Desmin (*DES*) maintains the overall structure of cardiomyocytes and cytoskeletal organization within striated muscle cells. Mitochondrial thioredoxin reductase 2 (*TXNRD-2*) is essential for mitochondrial oxygen radical scavenging. We describe a rare case of dilated cardiomyopathy (DCM) in an 18-year-old female with a heterozygous mutation involving both *DES* and *TXNRD-2* genes.

## Introduction

Dilated cardiomyopathy (DCM) is a cardiac disorder that can involve univentricular or biventricular enlargement with subsequent contractile dysfunction [1]. The prevalence of DCM is approximately 1:2500 in the general population and is estimated to result in 10000 deaths and 46000 hospital admissions in the United States per year [1,2]. DCM clinically manifests as a left ventricular ejection fraction of less than 40% with or without heart failure symptoms such as progressive dyspnea, ankle swelling, and exercise intolerance [1,2]. If not managed appropriately, it can lead to severe cardiac complications such as systolic and diastolic heart failure, arrhythmias, thromboembolisms, and cardiogenic shock [1-4]. DCM has multiple etiologies that involve structural damage and weakening of the myocardium yielding a clinical phenotype that is distinct from ischemic cardiomyopathies. These etiologies include myocarditis, autoimmune conditions, and drug-induced damage via alcohol abuse or other medications [1,2]. Notably, primary idiopathic DCM has been classified in several patients as well, in which genetic mutations play a significant role [1,2].

We report a rare case of combined mutation of two genes, involving *DES* and *TXNRD-2* causing DCM to discuss the currently available data on genetic mutation involved in dilated cardiomyopathy. Based on the literature review, this is the first case to describe two heterozygous gene mutations involving *DES* and *TXNRD-2* causing DCM. This article was previously presented as a conference abstract at the American College of Cardiology Together With World Congress of Cardiology (ACC Together With WCC or ACC.23/WCC) annual scientific meeting in New Orleans, Louisiana, on March 4, 2023.

## Case presentation

An 18-year-old female presented to the emergency department after an episode of syncope and reported episodic substernal atypical chest pain lasting for 10-30 minutes, occasionally radiating to the left chest. All other reviews of systems were negative and denied recent sick contacts or recent travel. The patient had a medical history of iron deficiency anemia and taking iron supplements. Denied history of alcohol use, illicit drug use, or tobacco use. Family history significant for cardiomyopathy of unknown etiology in her paternal grandmother and a congenital cardiac defect that was surgically repaired in her sister. 

The initial vitals were within normal range except for blood pressure of 90/60 mmHg. The patient was awake, and alert without any acute distress. Her initial electrocardiogram (ECG) showed a normal sinus rhythm with sinus arrhythmia. Initial laboratory results were within normal range except for mildly elevated B-type natriuretic peptide (BNP) of 157 pg/ml (reference range <100 pg/ml). Initial laboratory test results are listed in Table 1. 

**Table 1:** Initial laboratory test results. HCG: Human chorionic gonadotropin

Test	Result	Reference Range
Sodium	139	136 - 145 mmol/L
Potassium	4	3.5 – 4.8 mmol/L
Magnesium	1.8	1.6 - 2.6 mg/dL
Phosphorus	3.4	2.3 – 4.7 mg/dL
Chloride	107	98 - 107 mmol/L
Carbon dioxide	22	22 - 29 mmol/L
Glucose	77	77 - 100 mg/dL
Blood Urea Nitrogen	5	7 - 20 mg/dL
Creatinine	0.81	0.6 - 1.1 mg/dL
Total Protein	7.3	6.7 - 8.6 g/dL
Total Bilirubin	0.4	0.2 – 1.2 mg/dL
Aspartate transaminase	17	5 - 34 U/L
Alanine Transaminase	<6	0 – 37 U/L
Alkaline Phosphatase	48	40- 150 U/L
Calcium	9	8.4 – 10.4 mg/dL
Troponin T	<0.01	<0.04 ng/mL
B-Type Natriuretic Peptide	157	<100 pg/mL
Thyroid-stimulating hormone	1.2	0.27 - 4.2 uIU/mL
Creatinine kinase	99	29 – 168 U/L
Erythrocyte sedimentation rate	7	0 – 20 mm/hr
Carnitine, total	29	31 – 78 umol/L
Carnitine, free	27	22 – 63 umol/L
Carnitine, esterified	2	3 – 38 umol/L
Carnitine, esterified/free ratio	0.1	0.1 – 0.9
White Blood Cell Count	4.7	4 – 10 K/uL
Hemoglobin	12.2	11.2 – 15.7 g/dL
Hematocrit	37.4	34 - 45%
Platelet count	179	150 – 400 K/uL
HCG qualitative urine	Negative	Negative

**Figure 1:**
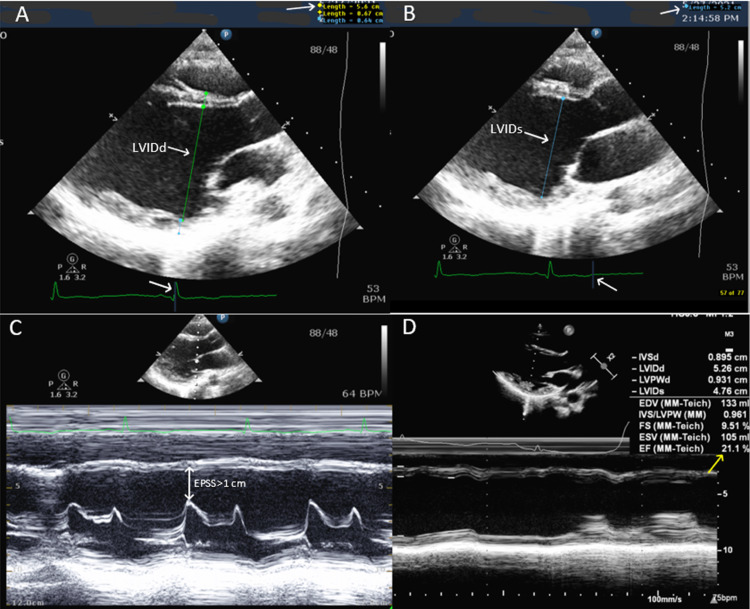
Transthoracic echocardiographic (TTE) images. Image A: shows left ventricular internal diameter end-diastole (LVIDd) 5.6 cm (white arrows) (reference range 3.5-5.6 cm). Image B: shows left ventricular internal diameter end-systole (LVIDs) of 5.2 cm (white arrows) (reference range 2.0-4.0 cm). Image C: shows E-point septal separation (EPSS) >1 cm suggesting a low ejection fraction. Image D: M-mode shows reduced ejection fraction (yellow arrow).

A transthoracic echocardiogram (TTE) (Figure 1) showed a dilated left ventricle with a reduced left ventricular ejection fraction of 28%. The patient was started on aspirin 81 mg daily, lisinopril 5 mg daily, and spironolactone 12.5 mg daily. The addition of other goal-directed medical therapy for heart failure was limited by hypotension. She was also experiencing postural tachycardia with a heart rate of up to 160 beats per minute and telemetry revealed several runs of non-sustained ventricular tachycardia. A dual-chamber implantable cardioverter-defibrillator was implanted, amiodarone 100 mg daily was added for non-sustained ventricular tachycardia, and ivabradine 5 mg twice daily for postural tachycardia. On hospital day 16, the patient was discharged on above mentioned medications.

She underwent extensive evaluation for the cause of heart failure including cardiac magnetic resonance, endomyocardial biopsy, and genetic study. The DCM was thought to be more likely genetic in origin due to the patient’s degree of compensation as evidenced by low BNP, negative troponin, no significant symptoms, non-sustained ventricular tachycardia, and evidence of late gadolinium enhancement on cardiac magnetic resonance imaging. Less likely diagnoses were prior infectious myocarditis such as coronavirus disease 2019 (COVID-19), chronic myocarditis (giant cell, lymphocytic, inflammatory), or arrhythmia-induced cardiomyopathy. Diagnostic tests performed during and after hospitalization are summarized in Table 2. 

**Table 2:** Diagnostic tests performed during and after hospitalization.

Test	Result
Electrocardiogram (hospital day 1)	Normal sinus rhythm with sinus arrhythmia.
Chest X-ray (hospital day 1)	Borderline enlarged heart and coarsened broncho vascular markings in the medial lower right lung.
Transthoracic echocardiography (hospital day 1) (Figure 1)	The left ventricle was moderately dilated and diffusely hypokinetic with an ejection fraction of 28%. Global longitudinal strain score of -9%. Normal right ventricular size with mildly diminished systolic function. Trivial mitral valve insufficiency. Trivial anterior pericardial effusion.
Cardiac magnetic resonance (hospital day 2)	Left ventricle: severely dilated with a left ventricular end-diastolic volume index (LVEDVi) of 144 ml/m^2^ (reference range: 50-96 ml/m^2^), severe diffuse global hypokinesis, and severely decreased global systolic function with an ejection fraction of 25%. Right ventricle: size and systolic function were normal. Extensively diffuse essentially circumferential subepicardial late gadolinium enhancement (LGE) involving basal and mid segments and to a lesser extent the apical segments, with extensive areas of additional patchy mid-wall LGE , most pronounced in the interventricular septum. On the T-2 weighted sequences, there was no definite associated increased T2 signal in the regions of LGE, which suggests that this may be more subacute/chronic myocarditis, rather than acute. Small pericardial effusion was noted.
Endomyocardial biopsy (hospital day 12)	Myocyte hypertrophy and patchy interstitial and replacement fibrosis with focal fatty replacement. No myocarditis or granuloma. Trichrome stain highlights fibrosis. Congo red stain for amyloid, as well as iron stain, was both negative.
Genetic cardiomyopathy panel (~2 months post-discharge)	Positive for two heterozygous gene variants of uncertain significance (VUS), one on desmin (DES) gene and one on thioredoxin reductase 2 (TXNRD-2) gene.

The patient presented for a follow-up 10 days post-discharge. She reported resolution of chest pain, no episodes of dizziness or syncope, palpitations, or implantable cardioverter-defibrillator shocks. The genetic test result was discussed with the patient and recommended a screening TTE and ECG for the patient’s parents and siblings. Over the period of ~10 months, the patient had a notable improvement in left ventricular ejection fraction to 42%.

## Discussion

Genetic factors in several individuals contributing to disease manifestation and DCM pathogenicity have been well characterized. These genetic defects are predominantly in the nuclear membrane, cytoskeletal, and sarcomere proteins that are crucial for proper contractile function, and these mutations have been identified collectively in 35% of DCM patients [2]. Typically, the alleles contributing to the DCM disease phenotype are inherited in an autosomal dominant pattern with variable expressivity, but there is growing evidence of X-linked and mitochondrial inheritance as well [5]. Truncating mutations in the titin (*TTN*) gene, which encodes for the titin protein, account for up to 25% of DCM cases [5]. The titin protein is the largest protein in the human body, comprising the predominant structural component of striated and smooth muscle sarcomeres [2,5].

Missense and truncating mutations on the short arm of chromosome 1 in the *LMNA* gene, encoding lamins A and C, are responsible for another 5-8% of total DCM cases [2,5]. These proteins are intermediate filaments that anchor nuclear chromatin and are important for cross-cellular transport [2,5]. *LMNA* mutations have also been implicated in muscular dystrophies and various arrhythmias, such as ventricular tachycardia and atrial fibrillation, which are common DCM complications [1,3-5]. Aberrations in genes encoding cardiac actin, dystrophin, desmin, and thioredoxin reductase 2 account for a small portion of genetic DCM cases [2,5]. 

Desmin, an intermediate filament encoded by the *DES* gene, plays a crucial role in the cytoskeletal structure of striated muscle [6]. Mutations in *DES* result in contractile impairment in cardiomyocytes and have been shown to contribute to familial DCM [6,7]. Our patient had a unique second mutation in the thioredoxin reductase 2 gene. Thioredoxin reductase is an enzymatic component of the thioredoxin, a major myocardial antioxidant system, responsible for scavenging reactive oxygen species and mitigating oxidative damage from ischemic injury and heart failure [8]. One previous case report highlighted the presence of a shared *TXNRD-2* mutation in a mother with peripartum cardiomyopathy and her infant son who died of DCM complications [9]. *TXNRD-2* gene has been linked to 1.3% of cases of DCM [9]. The prevalence of *DES* mutations related to DCM is between 1% and 2% [10]. The current patient demonstrates mutations in both *DES* and *TXNRD-2*, developed syncope, and markedly reduced left ventricular ejection fraction. These findings in conjunction with previous case reports demonstrate a potentially far greater contribution to DCM pathogenicity from these two genetic aberrations than previously thought and may inform early screening and management of carriers of these mutations. 

The evaluation of patients with symptoms suggestive of DCM relies on a combination of laboratory tests, imaging, and genetic studies to identify and treat underlying etiologies [1]. Currently, next-generation sequencing is widely used to detect pathogenic gene variants of DCM, and there is growing evidence of the efficacy of whole exome sequencing as well [5,11]. 

The 2018 Heart Failure Society of America (HFSA) genetic evaluation of cardiomyopathy guideline recommends the following screening: 1) clinical screening for cardiomyopathy in asymptomatic first-degree relatives, 2) asymptomatic first-degree relatives with negative findings should be rescreened at three to five years intervals beginning in childhood or any time symptoms or signs appear, 3) annual repeated clinical screening is suggested in first-degree relatives with abnormal clinical screening suggestive of or consistent with DCM [12]. The 2018 HFSA genetic cardiomyopathy guideline also recommends genetic and family counseling for all patients and families with cardiomyopathy and referral to centers experts in genetic evaluation and family-based management can be considered if sufficient expertise is not available locally [13].

Treatment of DCM involves the management of underlying etiologies and follows the standard protocol for heart failure treatment [1]. Medication management involves the use of goal-directed medical therapy (GDMT) for heart failure with beta-blockers, angiotensin-converting enzyme inhibitors, and angiotensin receptor blockers to treat patients with heart failure with reduced ejection fraction and maintain a euvolemic state. Patients who are in a more severe stage of the disease, as in the current manuscript, may need heart transplantation or device therapy with implanted cardioverter defibrillators to prevent sudden cardiac death [1].

## Conclusions

Dilated cardiomyopathy can present with severe and potentially life-threatening symptoms such as arrhythmias, cardiogenic shock, and thromboembolisms. This condition affects individuals across a wide age range and has several etiologies making it difficult to identify and treat at the early stages. Furthermore, there are several genetic variants in cytoskeletal and nuclear membrane proteins of cardiomyocytes that can contribute to disease pathogenicity. The current manuscript highlights an 18-year-old female patient with advanced dilated cardiomyopathy, with two genetic variants in *DES* and *TXNRD-2* that may have contributed significantly to disease progression. These two variants are likely more pathogenic than previously believed and may guide more effective genetic counseling and screening for patients with suspected dilated cardiomyopathy.
